# During natural vision, semantic novelty modulates fixation-related processing in primate cortex

**DOI:** 10.64898/2026.03.18.712708

**Published:** 2026-05-14

**Authors:** Vinay S Raghavan, Jens Madsen, Maximilian Nentwich, Marcin Leszczynski, Arnaud Falchier, Stephan Bickel, Brian E. Russ, Lucas C. Parra

**Affiliations:** 1 Department of Biomedical Engineering, The City College of New York, New York, NY, USA; 2 The Feinstein Institutes of Medical Research, Northwell Health, Manhasset, NY, USA; 3 Department of Psychiatry, Columbia University College of Physicians and Surgeons, New York, NY, USA; 4 Translational Neuroscience Lab Division, Center for Biomedical Imaging and Neuromodulation, Nathan Kline Institute, Orangeburg, NY, USA; 5 Cognitive Science Center, Jagiellonian University, Kraków, Poland; 6 Department of Psychiatry, New York University Grossman School of Medicine, New York, NY, USA; 7 Departments of Neurosurgery, Zucker School of Medicine at Hofstra/Northwell, Hempstead, NY, USA; 8 Departments of Neurology, Zucker School of Medicine at Hofstra/Northwell, Hempstead, NY, USA; 9 Lead Contact

**Keywords:** Natural Vision, Semantic Novelty, Scalp EEG, Intracranial EEG, Temporal Response Functions

## Abstract

We sample visual scenes with short gaze fixations separated by saccades. While low-level integration is known, semantic integration of foveal vision across multiple fixations remains unclear. We hypothesized that the brain responds to changes in semantic information from one fixation to the next, and therefore postulated a neural signal associated with semantic novelty for each saccade. Novelty was measured using a deep network on foveal vision. Novelty modulated frontal and occipital fixation-related potentials in human EEG during natural viewing of full-length movies (3.4x10^6^ saccades). Intracranial recordings in humans (9.0x10^4^ saccades) and non-human primates (3.3x10^4^ saccades) revealed broadband high-frequency activity modulations in ventromedial visual and frontal brain areas. This modulation was stronger for movies than static images, and frontal modulation preceded occipital modulation, suggesting top-down effects. This modulation of fixation-related activity with novelty suggests that foveal representations are integrated across saccades to construct scene representations during natural viewing in primates.

## Introduction

Humans and many other animals shift gaze via saccades followed by stable fixations to actively sample the visual environment with high-resolution foveal vision (Robinson, 2022). These eye movements evoke strong neural responses previously measured with scalp recordings (Kazai and Yagi, 1999; Degno and Liversedge, 2020) and intracranial recordings in humans (Bartlett et al., 2011; Barczak et al., 2019; Leszczynski et al., 2021, 2023) and non-human primates (Rajkai et al., 2008).

Prior evidence points to the integration of visual information across saccades. Peripheral input first aids target selection (Deubel and Schneider, 1996) and is then integrated with foveal input to maintain a continuous experience (Irwin and Gordon, 1998). Integration between foveal and peripheral vision occurs for attended, low-level features and object sizes (Wolf and Schütz, 2015; Valsecchi and Gegenfurtner, 2016). Whether foveal information is integrated across fixations remains unknown. High-level foveal-to-foveal integration would help construct a coherent scene representation from separate fixations. Semantic information plays a role in directing eye movements in static and dynamic scenes (Eisenberg and Zacks, 2016; Henderson and Hayes, 2017). Nevertheless, it remains unclear whether the brain integrates semantic visual information from a given sequence of fixations.

During a single fixation, the ventral visual stream extracts semantic information through a sequence of visual processing areas (Logothetis and Sheinberg, 1996), producing representations similar to deep vision models (Yamins et al., 2014; Yamins and DiCarlo, 2016; Lindsay, 2021). However, these computer vision models differ from biological vision in two key ways: they are limited to a single fixation, rather than multiple fixations, and they are often trained using supervised methods, i.e., with hand-labeled images.

Meanwhile, a revolution in AI has emerged avoiding training labels with self-supervised learning techniques compatible with natural vision. One particularly effective method learns robust representations using luminance fluctuations and crops of a larger image (Chen et al., 2020), comparable with successive fixations and variation in lighting. The embeddings learned by these deep networks capture semantic content by directly comparing representations between crops of the same image (Wen and Li, 2021).

We hypothesized a similar mechanism is at play in primate vision, whereby the semantic content of foveal vision is compared across fixations. If so, we expect semantic novelty to modulate neural activity associated with fixations while viewing static and dynamic scenes. We defined semantic novelty as the distance between deep embeddings of successive foveal fixation patches (Chen et al., 2020).

Saccade and fixation responses are modulated by saccade amplitude and low-level visual features (Dandekar et al., 2012; Ries et al., 2018; Madison et al., 2025). Amplitude, in particular, correlates with semantic novelty (Nentwich et al., 2023). Low-level visual features may also correlate with high-level semantics, jointly modulating responses (Lescroart et al., 2015; Greene and Hansen, 2020). These features relate to visual salience, and a range of features can predict gaze in scenes (Itti and Koch, 2001; Hayes and Henderson, 2021a). However, we are interested in how the brain responds to the integration of foveal content, rather than predicting saccade targets or modeling saccade control (Schiller and Tehovnik, 2005; Kümmerer and Bethge, 2023).

To address these possible confounds, we model the effects of saccade amplitude and low-level visual features alongside semantic novelty. We used a standard linear encoding model that accounts for correlated and delayed responses that may overlap between adjacent eye movements (Crosse et al., 2016; Holdgraf et al., 2017; Ehinger and Dimigen, 2019).

Our findings in human scalp potentials and intracranial recordings in human and non-human primates demonstrate a key role for semantic novelty in modulating fixation-related neural responses. By rigorously controlling for other features, we isolate a novelty response that could build scene representations across eye movements and tune the ventral visual system. This work provides evidence that semantic information is integrated between fixations.

## Results

We analyzed scalp and intracranial EEG (iEEG) in humans and non-human primates (NHP) across three free-viewing datasets of movies and images. These included human scalp EEG (79 participants), human iEEG (31 participants), and NHP iEEG (2 subjects). Most of these data (95.2%) have not been previously analyzed. Neural activity was measured via scalp potentials or broadband high-frequency amplitude (BHA).

**Figure 1.**
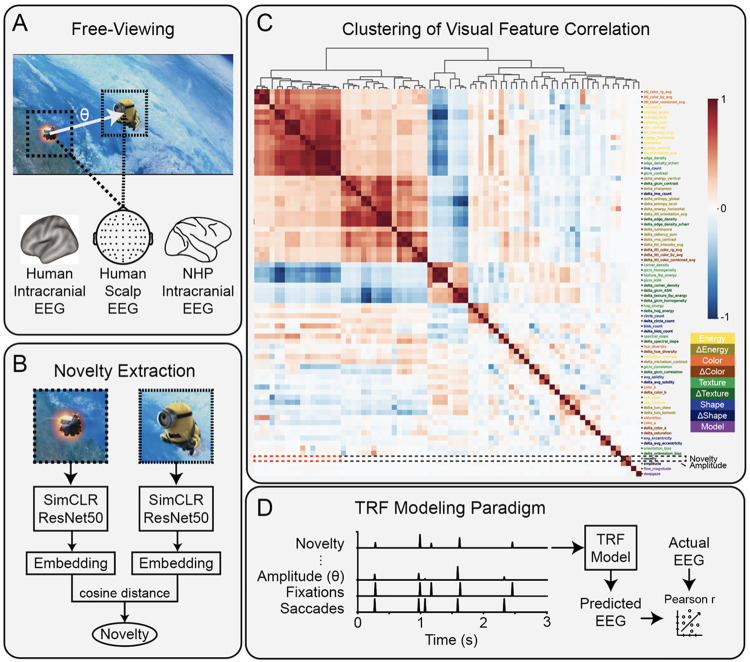
Examples of novelty computation and modeling paradigm. A) Illustration of a saccade, the amplitude (θ), and the fixation patches (5°) before and after the saccade, as measured alongside human scalp, human intracranial and NHP intracranial EEG. B) Novelty is defined as the cosine distance between semantic embeddings of the previous and current foveal fixation patches after embedding in a ResNet50 trained with SimCLR. C) Hierarchically clustered correlation matrix of visual features primarily related to energy, color, texture, and shape extracted from fixation patches from the human scalp EEG dataset. A large block of features are generally related to luminance or change in luminance (top-left). Novelty (black dash, bottom) was strongly correlated with only saccade amplitude (bottom-right) and weakly correlated with luminance-related features (red dash, bottom left). D) Saccades and fixations are represented as pulses in time, and features associated with each as scaled pulses. The model finds a temporal response function (TRF, i.e. a linear impulse response) for each feature that best predicts the EEG. Modeling performance is measured with Pearson’s correlation r.

To test the hypothesis that the brain responds to novelty across saccades, we computed novelty from each 5° foveal fixation patch and analyzed how this difference modulates fixation-locked neural activity. We measure the modulation of neural activity using a standard encoding model (Fig 1E)(Crosse et al., 2016; Holdgraf et al., 2017; Ehinger and Dimigen, 2019). This method learns a set of weights that linearly predict neural activity from a time-lagged version of the input. The model weights correspond to the impulse response of each feature, also referred to as the temporal response function (TRF). If adding a feature to the model significantly improves predictions of neural activity, i.e. there is a significant Δr, then we conclude the brain represents or “encodes” this feature.

To compute the semantic representation of each fixation patch, we passed the foveal patch through a ResNet50 trained with SimCLR (Chen et al., 2020) and extracted the final embedding (Fig 1C). This provides a high-level representation of each patch derived directly from a model trained with contrastive learning, i.e., to minimize distances between representations (embeddings) of different patches from the same image. Novelty is computed as the cosine distance between the embeddings of successive foveal fixation patches.

**Figure 2.**
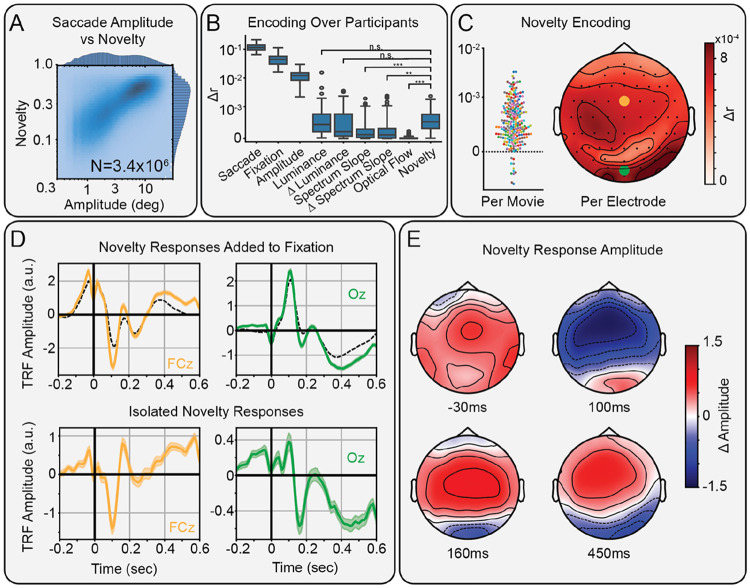
Modulation of scalp EEG by semantic novelty. A) Histogram of saccade amplitude vs novelty, showing a correlation over N=3.4 million saccades from 231 movie viewings in 79 participants. B) Box plots over participants of the increase in correlation between predicted and actual EEG, when adding each successive feature to the encoding model, shown in the order added. Novelty captures similar amounts of variance as luminance and Δ luminance, and significantly more variance than spectrum slope, Δ spectrum slope, and optical flow (n.s.: p>0.05, **: p<10^−2^, ***: p<10^−3^). C) Increase in correlation from adding novelty to the encoding model for each movie watched (left) and for each EEG electrode (right). Each dot is a viewing for a full-length film (90min - 120min). Color indicates the different movies (N=10). D) Average fixation-locked TRFs of electrodes FCz (left, yellow) and Oz (right, green). The additive effect of novelty (color) modulating the fixation response (dashed black; top).The isolated effect of novelty on fixation-locked responses (bottom). E) Topographic maps showing the modulation of components of fixation-locked responses by novelty at −30, 100, 160, and 450ms.

**Figure 3.**
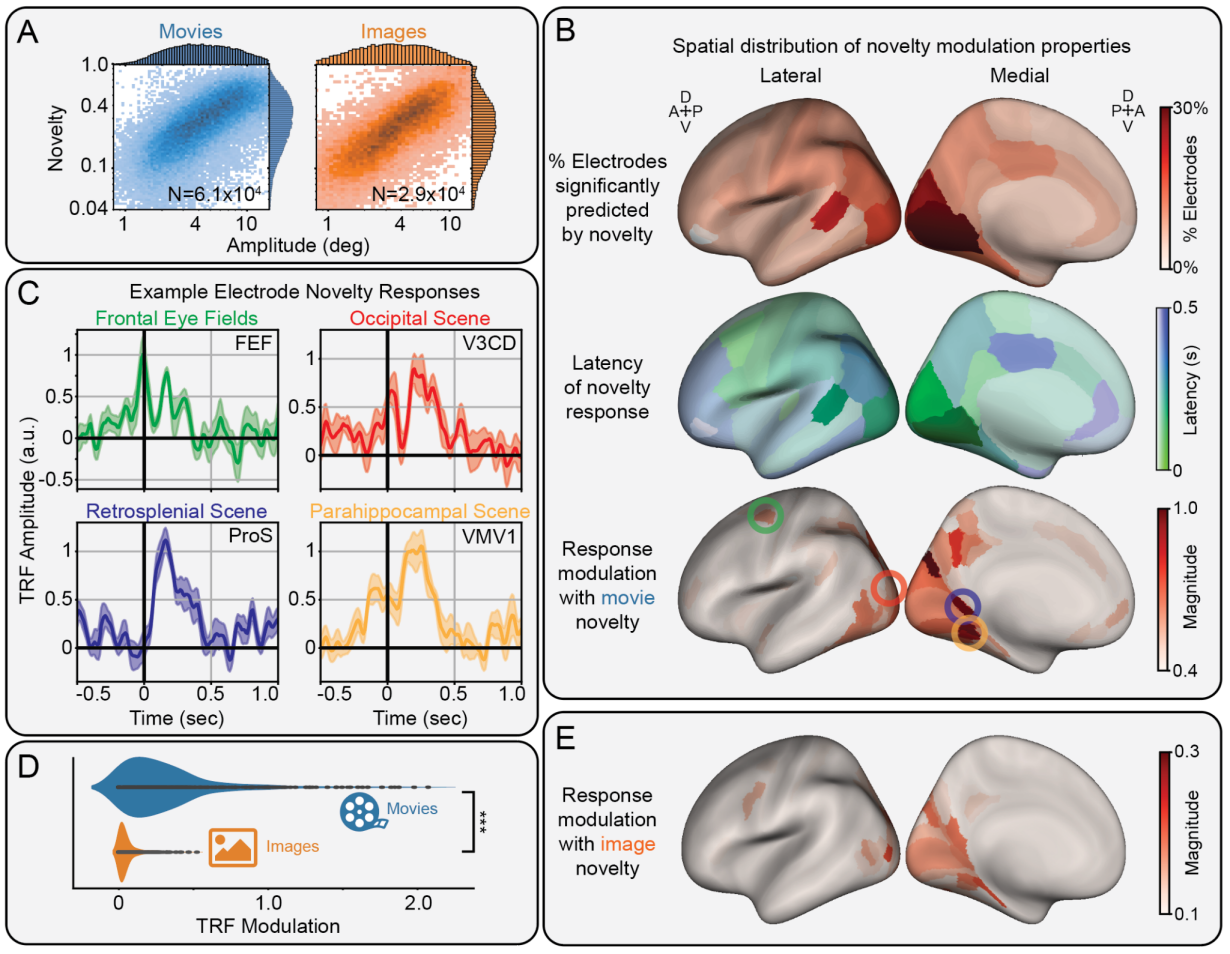
Modulation of human intracranial EEG by semantic novelty. A) Histogram of saccade amplitude vs novelty, showing a correlation over N=60,550 and N=28,797 saccades in the movie- and image-viewing datasets, respectively. B) Spatial distribution novelty modulation properties, including the percent of electrodes significantly encoding novelty (top), the average latency of novelty-responsive sites (center), and the maximum modulation for novelty-responsive sites during movie-viewing (bottom). C) Time course of novelty modulated fixation responses during movie viewing in selected brain regions, including the frontal eye fields (FEF), as well as the occipital (V3CD), retrosplenial (ProS), and parahippocampal (VMV1) scene areas (mean ± s.e.). D) Comparison of novelty modulation during movie and image viewing across all electrodes significantly encoding novelty. Modulation during image-viewing is significantly weaker across electrodes (***: p<0.001). E) Spatial distribution of the maximum modulation for novelty-responsive sites during image-viewing.

In all three datasets, we find that novelty is strongly correlated with saccade amplitude (Fig 2A, 3A, 4A). This is unsurprising, as more distant locations are more likely to capture semantically distinct elements in natural scenes. It is also possible that novelty is correlated with a number of visual features known to drive visual response. Therefore, we derived a range of low- and mid-level visual features from each fixation patch to determine their correlation with one another and with semantic novelty. We also included saliency features and optic flow to confirm they are unrelated (Dorr et al., 2010; Itti, 2005; Itti and Koch, 2000). While visual features generally show some correlation, semantic novelty appears to be an orthogonal measure to all features tested here (Fig. 1C). We only find smaller but significant correlations with several features related to luminance (Fig. S1). The analysis of fixation-related responses therefore regressed out effects of typical low-level features of natural scenes, including luminance, spectrum slope, and optical flow (Madison et al., 2025).

### Novelty locked to fixation broadly modulates human scalp EEG

We measured saccade- and fixation-related responses using TRFs in human scalp EEG. Saccades and fixations were coded as impulses to separate their neural contributions (Fig 1E). Saccade amplitude was aligned to saccade onset, while novelty and other visual features were aligned to fixation onset. This alignment best explained the variance in the EEG (Fig S2A). After accounting for saccade amplitude with non-linear responses (Dimigen and Ehinger, 2021), novelty significantly modulated fixation-locked but not saccade-locked responses (Fig S2C) confirming that novelty modulation is tied to visual processing. As expected, most variance is explained by the saccade and fixation regressors, with additional variance explained by the features that modulate these fixed responses (quantified as Δr in Fig. 2B). Notably, semantic novelty captures a similar amount of variance as luminance (t(78)=−1.06, p=0.59) and change in luminance (t(78)=0.72, p=0.59), and significantly more variance than spectrum slope (t(78)=5.55, p=1.5x10^−6^), change in spectrum slope (t(78)=3.50, p=2.3x10^−3^), and optical flow (t(78)=12.98, p=1.8x10^−20^; all p Holm-Bonferroni corrected), even with these features regressed out.

We measured how much novelty modulates fixation-locked responses. Across the 231 movie viewings (Fig 2C, left) and 64 scalp electrodes (Fig 2C, right), we found significant variance explained, particularly in frontal and occipital areas. Indeed, given the large number of saccades available (3.4x10^6^), all 64 scalp electrodes showed significant modulation (all Δr>3.7x10^−4^, p_boot_<0.01, FDR-corrected). The spatial distribution of variance explained on the scalp (Fig. 2C) rules out eye movement artifacts as the source of this modulation. Inspecting the TRFs for individual electrodes reveals how novelty modulates particular components of the fixation-related responses (Fig 2D).

To characterize the spatiotemporal effects of novelty, we plotted the spatial distribution of the novelty TRFs at local extrema (Fig 2E). These extrema were identified in clusters with permutation p < 0.01 by a non-parametric spatiotemporal cluster permutation test (Fig S2D)(Maris and Oostenveld, 2007). A fronto-central modulation precedes fixation onset by 30ms. At 100ms after fixation onset, we find the largest modulation of frontal areas. At 160 and 450ms after fixation onset, we find fronto-occipital dipoles, including occipital negativities.

These modulations match the sign of the baseline fixation-related potentials and thus represent an overall enhancement, rather than suppression, of the underlying neural response.

### Novelty modulates human intracranial EEG in scene-selective regions along the ventromedial visual stream

We analyzed fixation and saccade responses in the BHA of human iEEG in N=6328 electrodes with coverage over the entire brain (Fig S3). The same TRF approach was used to quantify the effects of novelty and separate it from other visual features and correlated saccade amplitude (Fig 3A). Electrodes containing saccadic spike artifacts due to extraocular muscle activity (Jerbi et al., 2009) were identified via hierarchical clustering of the correlation matrix of saccade TRFs and removed from further analysis (Fig S3)(Yeo et al., 2011). We found that 11% of the remaining sites (636/5782) significantly encoded novelty, primarily in occipital areas (p_boot_<0.05, FDR-corrected; Fig 3B, top).

The peak latency of novelty modulations across the cortex were quantified as the time of the absolute maximum of the signal, i.e., the highest peak or lowest trough. These peak latencies were interpolated onto each region of the Desikan-Killiany atlas (Fig 3B, center). This reveals two trends: an increasing latency along the ventro-medial visual stream from posterior to anterior areas, and an early frontal response, including activity around fixation onset, in the caudal middle frontal area, which includes the frontal eye fields (FEF).

We analyzed the modulations of electrodes significantly encoding novelty. On average, these sites favored enhancement (58%) rather than suppression (42%) of the BHA activity; however, the largest modulations consisted of enhancements (SFig 2C). The magnitude of the novelty modulation, defined as the maximum of the absolute value of the TRF, was interpolated onto the HCP-MMP atlas of the brain (Fig 3B, bottom). This reveals strong modulations in FEF, as well as along the ventromedial visual stream, particularly in scene-selective visual regions.

We plotted the novelty TRF of exemplar electrodes in each of these regions (Fig 3C). Consistent with the peak latencies (Fig 3B, center), we find FEF responds early, while visual areas respond later, generally after fixation. Strong enhancements are observed in each of the scene-selective visual areas, including the occipital, retrosplenial, and parahippocampal scene areas, corresponding to electrodes in HCP-MMP atlas regions V3CD, ProS, and VMV1, respectively.

An identical analysis was performed on the image-viewing dataset to determine if novelty modulation persists during image-viewing. We observed a significant (t(1,413) = 37.8, p = 2.6x10^−216^) decrease in the magnitude of novelty modulation across electrodes significantly encoding novelty in each dataset (Fig 3D). Plotting the modulation pattern from this image-viewing dataset on the brain reveals a similar spatial distribution to the movie-viewing dataset, but with a lower modulation magnitude (Fig 3E).

Overall, these results suggest semantic novelty enhances frontal activity before fixation onset, followed by strong enhancements along the ventromedial visual stream, especially in scene-specific areas, during movie-watching more than image-viewing.

### Novelty modulates NHP iEEG along the ventral visual stream during movie-watching more than image-viewing

**Figure 4.**
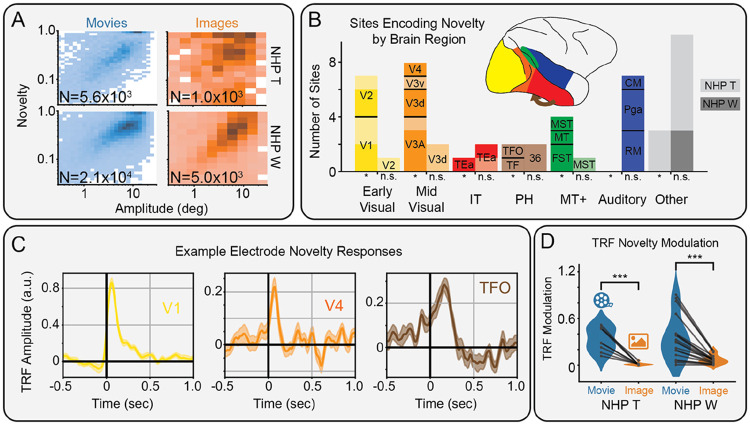
Modulation of non-human primate intracranial EEG by semantic novelty. A) Histogram of saccade amplitude and semantic novelty, during movie (left) and image viewing (right) for NHP T (top) and NHP W (bottom). B) Bar plot showing where electrodes were located, including those with (left bars) and without (right bars) significant (*: p_boot_ < 0.05, FDR corrected) modulation by novelty in NHP T (light shading) and NHP W (dark shading). C) Time course of novelty modulation in selected areas along the ventral visual stream, including area TFO. D) Modulations of novelty TRFs are significantly (***: p<0.001) stronger during movie- vs image-viewing.

Finally, we analyzed eye movement BHA responses during movie-watching in the iEEG recorded from two NHPs with 50 implanted sEEG contacts broadly covering temporal and occipital cortical regions. As before, we measured the effect of semantic novelty on neural activity beyond other low-level features, including saccade amplitude (Fig 4A). We find that 24/50 (NHP T: 9/23, NHP W: 15/27) sites are significantly modulated by novelty, particularly in occipital regions (Fig 4B). Measuring the type of modulation reveals a strong preference for enhancement (NHP T: 8/9, NHP W: 13/15) over suppression of BHA activity. This includes early, middle, and late responses along the ventral visual stream in V1, V4, and TFO, respectively (Fig 4C).

To examine the influence of a dynamic vs static stimulus, we compared the novelty modulation of neural responses during movie viewing with those during image viewing. We found that novelty modulation was significantly (NHP T: t(8) = 6.35, p = 2.2x10^−4^; NHP W: t(14) = 6.43, p = 1.6x10^−5^) reduced across novelty-modulated sites during the presentation of static images compared to movie clips (Fig 4D). This is consistent with our results in human iEEG responses. Overall, these results suggest that semantic novelty enhances activity along the NHP ventral visual stream following fixation onset during natural viewing of movies more so than images.

## Discussion

Our study demonstrates that semantic novelty enhances scalp EEG fixation-locked potentials and broadly enhances human and NHP iEEG BHA activity, particularly in scene-selective ventromedial visual regions. This spatial profile persists between image and movie viewing. Frontal areas, including frontal eye fields, show early modulation near fixation onset, suggesting anticipation of novelty, while occipital modulation occurs later. These findings indicate the visual system extracts foveal semantic information and integrates it across saccades to build a representation of the scene.

### Similarities to known EEG evoked response components

Fixed-gaze ERPs often parallel free-viewing fixation-related responses (Kamienkowski et al., 2012). Our data reveal modulations mirroring established novelty and prediction components during fixed gaze: The mismatch negativity (MMN), a response to stimulus change that occurs without attention (Pazo-Alvarez et al., 2003); the P3a, a fronto-central response reflecting automatic orienting to novel stimuli (Friedman et al., 2001); and the N400, a response to semantic incongruity shown in a range of senses and domains with varying spatial distributions (Kutas and Federmeier, 2011). We observe a P3a-like fronto-central positivity *before* fixation onset, consistent with the orienting process anticipating novelty before fixation. Next, we see fronto-occipital responses, including occipital negativities around 160ms and 450ms. While the frontal positivities appear to dominate the scalp maps, the occipital negativities observed more directly relate to those expected from novelty responses from the visual cortex, like the visual MMN and visual N400 (Barrett and Rugg, 1990; Astikainen et al., 2013).

### Early frontal modulation, late occipital modulation

We find modulations in frontal areas near fixation onset. Early frontal modulation suggests novel semantics are processed in the periphery or anticipated from context, aligning with work showing novelty signals during the *preceding* fixation (Coco et al., 2020). Conversely, late modulation in early visual areas suggests top-down feedback, where foveal areas respond to peripheral inputs after a delay (Stewart et al., 2020). The delayed timing of this effect is consistent with the timing of integration of peripheral signals with subsequent foveal input (Ramezani et al., 2019). Modulation of early areas with semantic novelty is consistent with fMRI studies showing that high-level object category (extracted from peripheral vision) is fed back to the foveal retinotopic visual cortex (Williams et al., 2008).

### Novelty modulation in scene-selective regions indicates the role of semantics

Our results revealed particularly strong modulation with novelty in the parahippocampal, retrosplenial, and occipital scene areas in humans (Epstein and Baker, 2019) and area TFO in NHPs also associated with scene processing (Kornblith et al., 2013). This common modulation suggests these streams are conserved across species (Vinken et al., 2025), potentially including the ventral novelty stream (Weierich et al., 2010; Kafkas and Montaldi, 2018). Unlike past work in fMRI (Lescroart et al., 2015), this semantic novelty measure captures variance above and beyond low-level features, similar to (Greene and Hansen, 2020). The activation of scene-selective regions indicates that saccades to semantically novel content help build up scene representations. This is consistent with prior work showing the importance of saccade targets for transsaccadic integration in natural scenes (Henderson and Hollingworth, 2003) and the role of oculomotor cues for integration in scene-selective regions (Golomb et al., 2011). It also aligns with gaze prediction work indicating that eye movements are drawn to regions semantically coherent with the scene (Hayes and Henderson, 2021b).

### Stronger responses to movies vs images

Novelty responses in movies were stronger than in images throughout the cortex, aligning with existing studies showing stronger responses to dynamic vs static stimuli for faces,(Pitcher et al., 2011) bodies and objects (Küçük et al., 2024), as well as scenes (Korkmaz Hacialihafiz and Bartels, 2015). This is typically found in brain areas sensitive to motion, such as integrated motion in area MT/V5 (Born and Bradley, 2005; Hedges et al., 2011), self-motion and optic flow in MST (Wild and Treue, 2021), and “biological motion” in STS (Puce and Perrett, 2003; Blake and Shiffrar, 2007), but rarely is it found in the ventral visual stream (Pitcher et al., 2019). The modulation we observed was specific to semantic novelty. We propose two explanations: First, movies increase engagement and attention to the stimulus, increasing evoked activity. Second, our novelty measure based on static image crops may underestimate the actual novelty due to dynamics in the foveal input during fixations, leading to an overestimate of modulation with novelty during movies.

### Possible role of enhancing responses with novelty

We found that novelty primarily enhanced fixation responses. While probabilistic population coding suggests expected stimuli should evoke stronger responses (Ma et al., 2006; Beck et al., 2008), most temporal prediction models argue that they should elicit weaker responses (Summerfield and de Lange, 2014). In V1, expected stimuli cause weaker fMRI response but they can be more readily decoded (Kok et al., 2012). This has been interpreted as “sharpening” the representation for expected stimuli (de Lange et al., 2018). The theory of “predictive coding” argues that “error” units compare top-down prediction with bottom-up input and respond strongly when there is a mismatch (de Lange et al., 2018). The theory often also argues that the error signal is propagated forward along the visual processing hierarchy and serves as the primary “encoder” of the sensory input (Millidge et al., 2022). However, the biological realism of this error-coding mechanism is not universally accepted (Walsh et al., 2020; Solomon et al., 2021; Westerberg et al., 2024). More widely accepted is the notion that prediction errors are relevant for learning (Roelfsema and Holtmaat, 2018).

### Error signal as a potential tuning signal

This work was motivated by recent progress in machine learning that shows that vision models can be trained without labeled data, but simply by predicting representations of one image crop from the next. The concept has been incorporated in a “joint embedding predictive architecture” (LeCun, 2022; Assran et al., 2023), which has inspired a number of powerful computer vision models. There, the error in predicting the upcoming embedding is the training signal to tune the network. If a similar mechanism is at play in the brain, we expected ubiquitous novelty signals, which indeed we found. We do know that vision remains adaptive throughout life (Webster, 2015). Indeed, there is direct evidence that prediction between peripheral and subsequent foveal vision does serve to calibrate size perception (Valsecchi and Gegenfurtner, 2016). More generally, we speculate that semantic novelty, which can be thought of as a prediction error, may serve to tune the ventral visual system engaged in object categorization.

### Limitations

Our measure of novelty did not take peripheral vision into account, which is known to influence foveation targets (Stewart et al., 2020). Psychophysics research shows that integration of current peripheral vision with upcoming foveal vision is limited to attended targets (Deubel and Schneider, 1996; Irwin and Gordon, 1998). Neural recordings show neurons responsive to foveal vision respond, shortly before a saccade, to the stimulus already visible in peripheral vision at the location of the upcoming fixation (Duhamel et al., 1992; Neupane et al., 2020). Evidence for semantic processing of peripheral vision also comes from scalp recordings with neural responses to low-level features of the target *prior* to the upcoming fixation (Ehinger et al., 2015; Buonocore et al., 2020; Stankov et al., 2021a). Future studies should explore alternative measures of novelty that incorporate peripheral vision.

Finally, we tested for correlations between novelty and several visual features and specifically controlled for luminance, spectrum slope (Hansen et al., 2011, 2012; Madison et al., 2025) and optical flow features (Nentwich et al., 2023; Yao et al., 2024). However, deep representations of the image from different layers of the network may also relate to this measure. Future work may explore if intermediate representations of candidate deep-network better match population activity observed at particular stages of the primate visual processing streams (Yamins et al., 2014), and linking timing to various levels of processing (Cichy et al., 2014; Contini et al., 2017).

## Conclusion

Our findings provide compelling evidence that the visual system computes a novelty of the semantic content of fixations. This novelty is reflected in the modulation of fixation related responses across multiple brain regions and in both humans and non-human primates. By rigorously controlling for saccade amplitude and low-level features, we isolate a purely semantic novelty that likely serves to tune the ventral visual system, including scene-specific areas. This is consistent with our postulate that subsequent fixations integrate semantic information to build a representation of the scene. These results challenge traditional models of vision that treat each fixation as an independent snapshot and highlight the importance of considering the temporal dynamics of eye movements and the predictive nature of visual processing.

## Methods

### Participant details

The human scalp dataset comprised 79 participants (41 female), aged 18-37 years (M = 23.43, SD = 4.67), whose data met inclusion criteria and were included in the analyses. All procedures were approved by the Institutional Review Boards of the City University of New York, and all participants provided informed consent.

The human intracranial dataset comprised 31 patients (age 18-59, mean 38, 16 female) with a total of 8522 electrodes implanted. All patients are included in the image dataset, and a subset of 23 patients (6328 electrodes) is included in the movie dataset (age 19-58, mean 38, 11 female). Patients were implanted with depth, grid, and/or strip electrodes for clinical treatment of drug-resistant epilepsy at Northwell Health (NY, USA). Four patients were implanted twice at different times, and sessions were recorded again from these patients to capture the new electrode coverage. Three re-implants are included in the movie dataset. The study was approved by the institutional review board at the Feinstein Institute for Medical Research, all clinical investigation was conducted according to the principles expressed in the Declaration of Helsinki, and all patients gave written informed consent before electrode implantation.

The NHP dataset included 2 rhesus macaques (Macaca mulatta; 2 female, age 4-9). All procedures were approved by the Institutional Animal Care and Use Committee of the Nathan Kline Institute and were carried out in accordance with NIH standards for work involving non-human primates. The two animals, NHP T and NHP W had a total of 27 and 23 electrodes implanted in cortical or subcortical regions, respectively.

### New versus existing data

In total, judging by duration, 95.2% of the data in this study have not been previously analyzed. The fraction of the human iEEG data that have been previously analyzed (79.7%) did not study fixation-related responses for visual features as we have done here. Specifically, the four distinct datasets are as follows: New human scalp data with movies (238 viewings, 22,000 min total). Existing human intracranial data with movies (23 patients, 1063.7min total, with 23 previously analyzed in (Nentwich et al., 2023); 22 in (Nentwich et al., 2025); 21 in (Mishra et al., 2025); 8 in (Leszczynski et al., 2023)), largely new human intracranial recordings with images (9 of 31 patients, 93.6min of 387.6min previously analyzed in (Leszczynski et al., 2023)), and new non-human primate intracranial with movies and images (movies 133min+73min; images: 56min + 13min). Separate data release papers are forthcoming for these datasets.

### Experimental materials and procedure

Each participant in the human scalp movie dataset watched between 1 and 10 full-length movies (1.5–2 h) selected from the following set: *The Big Sick, The Peanut Butter Falcon, Whiplash, Room, Me and Earl and the Dying Girl, The Tomorrow Man, Dom Hemingway, Life After Beth, Woodshock*, and *The Comedian*. After applying exclusion criteria, 79 participants contributed a total of 244 participant–movie viewings across these 10 movies, with participants watching an average of 3.09 movies (median = 2; range = 1–10). Participants were positioned in a comfortable reclinable chair with a neck rest and wore a headrest to constrain head movement. Participants were instructed to watch the movie as they would normally watch a movie. After viewing, participants answered questions about the movie (not used in this study).

Each participant in the human intracranial movie dataset watched 29.3 to 43.7 minutes of videos (mean ± sd: 40.9 ± 4.7 minutes), for 1063.7 minutes total. The videos included a 10 minute clip of the animated movie Despicable Me (English-language), a non-overlapping 10 minute clip of Despicable Me (Hungarian-language), a 4.3 minute clip of the animated short film The Present (English-language), and three 5 minute clips of documentaries of macaques (no sound), all used previously (Russ and Leopold, 2015; Russ et al., 2021; Nentwich et al., 2023; Telesford et al., 2023).

Each participant in the human intracranial image dataset viewed 6.1 to 28.1 minutes of images (mean ± sd: 12.5 ± 3.7 minutes) for a total of 387.6 minutes. 80 unique images were presented, each viewed no more than once. The image content ranged from animals, people, and food to animated movies, vehicles, and toys.

Each NHP watched the same video clips and viewed the same images as the human intracranial participants. Each video was watched multiple times by each NHP. NHP T watched each of the three macaque documentaries 4, 5, and 5 times, for a total of 133 minutes. NHP W watched each of the three macaque documentaries 3, 5, and 5 times, and each of the other videos twice each (Despicable Me - English, Despicable Me - Hungarian, The Present), for a total of 73 minutes. NHP T and W both viewed each image several times, for totals of 56 and 13 minutes, respectively

### Data acquisition

Human scalp EEG was recorded at 2048 Hz using a BioSemi ActiveTwo system with 64 scalp electrodes placed according to the 10-10 international system. Four electrooculogram (EOG) electrodes were placed above, below, and lateral to the eyes to capture ocular artifacts. Eye position (single eye) was recorded using the SR Research EyeLink 1000 system with a 35-mm lens at a sampling rate of 500 Hz allowing free head movements. A standard 9-point calibration was followed by manual verification. Head movement was constrained by subjects wearing a headrest as they sat in a reclined comfortable chair. Saccades and blinks were detected using the device’s built-in algorithm.

Intracranial EEG from human participants was recorded from stereoelectroencephalography depth electrodes, subdural grids, and/or subdural strips (Ad-Tech Medical Instrument Corp., Oak Creek, WI, USA; Integra LifeSciences, Princeton, NJ, USA; PMT Corp., Chanhassen, MN, USA). Subdural grid/strip contacts were 3-mm platinum disks with 10-mm intercontact spacing. Depth electrode contacts were 2-mm cylinders with 0.8-mm diameter and 4.4- or 2.2-mm intercontact spacing. Intracranial electrode signals were referenced to a subdermal electrode or subdural strip. Data were sampled at 3 kHz (16-bit precision, range ± 8 mv, DC) on a Tucker-Davis Technologies data processor (TDT, Alachua, FL, USA), or at 1KHz an XLTEK Quantum Amplifier (Natus Medical) for 2 of the patients in the image dataset. Gaze position from both eyes was recorded simultaneously with a Tobii TX300 eye tracker (Tobii Technology, Stockholm, Sweden) at 300 Hz allowing free head movements. The eye tracker was recalibrated after each video to prevent drift. Parallel port triggers were sent from the stimulus PC to the eye tracker and the data processor to align the data streams. Custom scripts for movie and image presentation with Psychtoolbox (version PTB_Beta2014-10-19_V3.0.12; Gstreamer version 1.10.2) and for collecting eye tracking data with the Tobii SDK were implemented in MATLAB (2012b, Windows 7). For additional accuracy in the alignment of streams, we recorded timestamps at the onset of each video frame with the clock of the eye tracker.

All procedures were approved by the IACUC of the Nathan Kline Institute for Psychiatric Research. Two female rhesus macaque (*macaca mulatta*) monkeys (at time of recordings: NHP-T: 4.5 yrs ~4.5-5.25 kg; NHP-W 9 yrs, 4.5-6.0 kg) participated in the current experimental protocol. All experimental tasks were run using SR Research Experiment Builder (SR Research Ltd., Mississauga, Canada).

Prior to neural recordings, animals were implanted with MRI-compatible headposts and trained to fixate visual targets for reward, in order to calibrate their eye positions for future studies. Both animals were then implanted with Spencer sEEG electrodes (Ad-tech, USA) each with 12 contacts (5 mm spacing) covering one hemisphere of the brain. We obtained structural MRIs (Siemens Trio 3T: 4-6 MPRAGE T1w images) prior to implantation, which were used to target the positioning of the sEEG probes. Electrodes were implanted through a craniotomy over the occipital lobe and extended toward the anterior portion of the brain using a stylet till they reached their target location. The first animal, NHP-T, was implanted with three 12 channel sEEG probes extending from the occipital lobe (V1) towards the midbrain (putamen), the anterior ventral temporal cortex (area TE), and the frontal lobe (area 13a&b). The second animal, NHP-W, was implanted with four 12 channel sEEG probes extending from the occipital lobe (V1) towards the superior temporal cortex (rostromedial cortex), the anterior ventral temporal cortex (area TEm), the midbrain (LGN), and the frontal lobe (striatum). Following implantation, the final electrode positions were confirmed with a second MRI imaging session.

Following a 6-8 week recovery period, we began recording neural data while the animals participated in a free-viewing paradigm. Each session, the animals performed a 3 point calibration. Eye position was sampled using EyeLink 1000 system (SR Research Ltd., Mississauga, Canada) at 1000Hz. After calibration the animals participated in blocks of movie viewing, where they were allowed to freely view 4-10min movies. We recorded local field potentials during movie watching using a NeurOne EEG system (Bittium, Oulu, Finland) at 40kHz.

### Intracranial electrode localization

In the human intracranial dataset, the electrode arrays each contained multiple contacts, which were identified using the iELVis MATLAB toolbox (Groppe et al., 2017). All participants received a preoperative T1-weighted 1mm isometric scan on a 3T scanner. Tissue segmentation and reconstruction of the pial surface were performed with the FreeSurfer package. Postoperative CT scans were acquired and coregistered to the FreeSurfer reconstruction. Contacts were semi-manually localized using the BioImage Suite (version 3.01)(Papademetris et al., 2006). All contacts were then coregistered to the FSAverage brain for visualization and assignment to anatomical atlases (see *Human intracranial EEG brain plots*).

In the NHP intracranial dataset, depth electrodes were visualized with a set of post-operative T1-weighted .6mm isotropic scans. The images were registered to each other using AFNI’s 3dAlineate command (Cox, 1996), averaged, and aligned to NMT Atlas space (Seidlitz et al., 2018; Jung et al., 2021). Contact locations were then determined based on their location relative to the CHARM and SARM hierarchical atlases (Seidlitz et al., 2018; Hartig et al., 2021; Jung et al., 2021).

### Neural data pre-processing

The human scalp EEG data were band-pass filtered (0.5-64 Hz), downsampled to 100 Hz, and manually inspected for noisy channels. Interpolation of bad channels was conducted using neighboring electrodes in a 3D-projected coordinate space. Robust Principal Component Analysis (Robust PCA) was applied for artifact removal in both EEG and EOG channels. Eye-movement artifacts were further removed via linear regression of the EOG channels from the EEG data. Residual outliers exceeding ±4 interquartile ranges were replaced by interpolation using neighboring electrodes within ±40 ms.

Both human and NHP intracranial datasets had the same preprocessing steps, all implemented using naplib-python (Mischler et al., 2023). The raw data were resampled to 600 Hz, and a 4th-order Butterworth high-pass filter with a cutoff of 0.5 Hz was used to remove DC drift. Data were re-referenced using a local average scheme whereby each electrode was referenced relative to the average of its nearest neighbors, which depend on the physical configuration of each electrode array. Line noise at 60 and 120 Hz was removed with order 501 FIR filters with 1Hz bandwidth applied forwards and backwards for zero phase shift. The data were then filtered into the high gamma band/broadband high-frequency (70-150 Hz), which reflects local population activity that is related to, but dissociable from, neuronal spiking (Leszczyński et al., 2020). To obtain the broadband high-frequency amplitude (BHA), we used the naplib-python toolbox to filter the data into 8 frequency bins logarithmically spaced between 70 and 150 Hz using Chebyshev Type 2 filters. The envelope of each band was obtained by taking the magnitude of the analytic signal obtained via the Hilbert transform. All eight frequency band envelopes were summed, and the resulting signals were resampled to 100 Hz and z-scored.

### Eye movement detection

In the human scalp and NHP intracranial datasets, eye movements were recorded with SR Research EyeLink hardware, and the default parameters were used for eye movement detection. Specifically, saccades were detected as periods in which angular velocity and acceleration were greater than 30°/s and 8000°/^2, respectively.

In the human intracranial datasets, eye movements were recorded with the Tobii TX300, so additional pre-processing was needed to detect saccades. A 20th-order median filter was used to smooth gaze position data. This reduces high frequency noise that can lead to false positive saccades. Angular velocity was computed for easier interpretation of saccade amplitude. Saccades were labeled as samples of eye velocity greater than 2 standard deviations from the average. Short adjustments of gaze position after the saccade are merged into the saccade using a morphological closing operation with a kernel size of 5 samples so that the overshoot is not detected as a separate saccade. The fixation onset corresponds to the first sample after which eye velocity drops under the 70th percentile, computed from velocity values within 33 ms before and 120 ms after saccade onset. The eye tracker provides labels for data quality when the gaze was not detected, e.g., during eye blinks. Saccades within 83 ms of samples with low data quality are removed, because this time period included gaze adjustments after blinks We also remove saccades following fixations less than 110 ms long, which are on the lower range of typically observed intersaccade intervals (Otero-Millan et al., 2008).

Saccade amplitude was calculated for all datasets by first determining the change in horizontal (x) and vertical (y) gaze angles between the saccade's onset and offset. The amplitude was then defined as the 2-norm (Euclidean distance) of this change vector.

### Fixation patch and feature extraction

For each fixation, we extracted an image patch subtending 5 degrees of the visual field, roughly corresponding to foveal vision. A patch size of 5 degrees corresponds to different patch resolutions depending on the physical position of the participant and monitor. In the human scalp, human intracranial, and NHP intracranial datasets, patch resolutions were defined as 100x100, 200x200, and 200x200, respectively. These were computed based on the screen resolution, screen size, and distance between the participant and the screen.

We extracted 37 distinct features related to each fixation patch to test if any of them correlated with semantic novelty (Fig. 1C, Table S1). In addition to the feature value at each patch, we also measure the difference in feature value across the saccade, analogous to how semantic novelty is measured. From the CIE L*a*b* representation of the image patches, we computed average luminance, luminance skew, luminance kurtosis, average a* channel color, and average b* channel color. From the HSV representation, we computed average saturation and hue diversity. From the grayscale image, we computed the Michelson contrast, RMS contrast, sharpness, vertical energy, horizontal energy, orientation bias, spectrum slope, global entropy, average local entropy with a 7x7 kernel, and energy in the histogram of oriented gradients. We used the grayscale image to extract additional texture and shape features, including local binary pattern energy, Scharr edge density, Sobel edge density, Hough line count, and Hough circle count. We also extracted gray-level co-occurance matrix (GLCM) features, including contrast, homogeneity, correlation, and angular second moment. We defined blobs by thresholding each patch at the mean grayscale value and performed blob analysis, computing the number of blobs, average eccentricity, and average solidity. Finally, we computed salience features, including the intensity (6 maps), color (6 RG, 6 BY maps), and orientation (4 orientations, 6 scales) salience features described previously, as well as DeepGaze III saliency of the fixated location in the 100 to 400ms preceding saccade onset (Itti and Koch, 2000; Kümmerer et al., 2022). We also extracted optical flow features (Nentwich et al., 2023; Yao et al., 2024).

Ultimately, we modeled 3 low-level visual features related to each fixation patch: luminance, spectrum slope, and optical flow. The average patch luminance was computed as the mean of the perceptual lightness channel in CIE L*a*b* colorspace (Bradski, 2000). To extract the spectrum slope, we computed the Fourier transform of the lightness channel, defined 8 logarithmically-spaced frequency bins between 0.1-10 cycles/degree, computed the mean magnitude of the patch spectrum in each bin, and extracted the negative slope of the linear regression line best fitting the log amplitude vs log frequency plot (Hansen et al., 2011; Madison et al., 2025). In addition, we included as features the changes in luminance and spectrum slope between successive fixation patches, computed simply as the differences (Madison et al., 2025).

To extract optical flow, we first computed optical flow versions of each video using the Farneback method. Each video was saved using float16 data with a 10x downsample to the width and height to reduce storage overhead and information redundancy. Next, for each saccade, we computed the preceding optical flow as the mean Farneback optical flow within the patch in the −250ms to −50ms preceding saccade onset, as visual change in this period often relates to upcoming eye movements (Saslow, 1967).

Semantic novelty across saccades was computed using a convolutional neural network trained using the Simple framework for Contrastive Learning of visual Representations algorithm (SimCLR)(Chen et al., 2020). In all datasets, fixation patches were upsampled to 224x224 for input to the network using 3rd order spline interpolation. Representational embeddings of the pre- and post-saccadic patches were extracted from a ResNet50 trained with SimCLR, and novelty is defined as the cosine distance between these embeddings. A large cosine distance between embeddings corresponds to semantically unrelated image patches and thus, a high semantic novelty. This process was identical to previous work (Nentwich et al., 2023).

### Temporal response function encoding models

To determine how novelty modulates neural responses around eye movements, we used linear encoding models to predict neural activity at each electrode as output, using a time-lagged representation of eye-movement events and features as input (Holdgraf et al., 2017). The weights of these models are often called temporal response functions (TRFs), as the weights correspond to the brain’s responses that are linearly predictable from the input (Crosse et al., 2016). After these weights are learned, the model fit can be tested in a cross-validated manner to determine how well the model's predictions of neural activity match the true neural activity using Pearson correlation.

#### Banded ridge regression

Ridge regression is often used in encoding models because regularization helps prevent overfitting due to limited or noisy data. Many previous studies use the same regularization parameter for the entire combined feature space. However, it is not clear that this is the best solution, given that feature spaces with different units, different numbers of dimensions, different degrees of correlation, different degrees of sparsity, and/or different degrees of predictive power will require different regularization values to prevent overfitting (De Heer et al., 2017). Therefore, we use banded ridge regression to set a separate regularization value, λ, for each feature space (Nunez-Elizalde et al., 2019; Dupré La Tour et al., 2022). To do so, a cross-validation procedure is used, involving a search over each set of λ values for each feature space. This is done in a pre-specified order such that any shared variance will be attributed to the feature added first. This is implemented by sweeping λ_1_ for the first feature, identifying λ_1_ that maximizes the average cross-validated correlation between the true and predicted neural responses, and freezing λ_1_ for the first feature. Then, the next feature is concatenated to the input, and now λ_2_ is swept for the second feature while holding λ_1_ constant to identify the λ_2_ that maximizes the prediction correlation. This process is repeated for each feature. This sequential approach assures us that any variance explained by an additional feature is not due to correlations with previously added features. We have used this same procedure in past work (Raghavan et al., 2023; Raghavan and Parra, 2025).

#### B-spline non-linearities

The influence of saccade amplitude on EEG activity is not strictly linear (Ries et al., 2018); however, TRFs only measure responses linearly predictable from the stimulus. To handle this, we use cubic B-splines to perform spline regression, similar to the *Unfold* toolbox (Ehinger and Dimigen, 2019). This approach models the predictor values using an expanded basis set, allowing for certain non-linearities to be learned, such as those observed with saccade amplitude (Dimigen and Ehinger, 2021). Here, we modeled saccade amplitude using five B-splines with knots defined based on the quantiles of each feature in each dataset. This allows us to measure any non-linearities in the modulations due to saccade amplitude across the range of values. The effect of novelty on saccade vs fixation responses was also tested using B-spline non-linearities (SFig 1C); however, adding a non-linearity to novelty did not considerably improve neural predictions, so it was ultimately not used. Similar to past work, low-level visual features were modeled linearly (Madison et al., 2025).

#### TRF modeling of feature hierarchy

All models were trained using the sklearn Ridge model solved via Cholesky decomposition and used cross-validation (CV) within participants to prevent overfitting (Pedregosa et al., 2011). The movie datasets used leave-one-trial-out CV to avoid concatenation of discontinuous data. Trials are movie chapters in the human scalp dataset and movie segments in the human and NHP intracranial datasets. The image datasets used a 5-fold CV to ensure sufficient data was present in each fold.

The human scalp dataset used a time range of −0.2 to 0.6 seconds to capture the main components of each response, while limiting the extent to avoid unnecessary computational overhead due to the large size of the dataset. Fifteen regularization values, λs, were tested on a logarithmic scale from 10^−2^ to 10^5^. The human and NHP intracranial datasets used a time range of −0.5 to 1.0 seconds to capture the full BHA response of electrodes with a wide range of latencies. Ten regularization values, λs, were tested on a logarithmic scale from 10^0^ to 10^3^. Regularization was applied by dividing the input features by their corresponding λ, as this is equivalent to multiplying the regularization term by λ (Nunez-Elizalde et al., 2019).

To measure the effect of novelty on neural responses invariant to other low-level features, we used banded ridge regression to model each feature hierarchically. We measured feature encoding in the following order: saccade onset, fixation onset, saccade amplitude, luminance, change in luminance, spectrum slope, change in spectrum slope, preceding optical flow, and novelty. This ensures that all effects of novelty can be reliably attributed to novelty directly, rather than any of the low-level features.

#### Statistical testing of model predictions and TRF weights

Statistical tests performed to test whether novelty significantly modulated neural responses depended on the dataset. In the human scalp dataset, we first tested whether novelty was significantly encoded in neural activity using the hierarchical bootstrap of the encoding results, i.e., prediction improvement Δr, over participants and chapters (N_boot_ = 10^4^)(Saravanan et al., 2020). To confirm the TRF weights representing the novelty response showed significant differences from zero, we performed a non-parametric spatiotemporal cluster permutation test utilizing the adjacency matrix of the BioSemi64 montage and N=10^4^ permutations (Maris and Oostenveld, 2007). This revealed four clusters with permutation p<0.05 (SFig 2D), which include peaks at the noted time points (Fig 2E).

In the human and NHP intracranial datasets, we tested whether novelty was significantly encoded at each electrode. To do so, we bootstrap the encoding results over trials and apply false discovery rate (FDR) correction to the resulting values (Benjamini and Hochberg, 1995; Saravanan et al., 2020). Only electrodes with p_boot_ < 0.05 with FDR correction are considered significant. To test the significance of the TRF peaks used to compute peak magnitude and latency, we bootstrap the TRF values at their peak magnitude, take all p-values from the TRF peaks, and apply FDR correction to this set of p-values. Only electrodes that have a peak TRF value with p_boot_ < 0.05 with FDR correction were shown in magnitude and latency plots.

#### Comparing saccades and fixations in human scalp EEG

Given that related work differs between the usage of saccade- and fixation-locked brain activity (Ries et al., 2018; Stankov et al., 2021b; Nentwich et al., 2023; Madison et al., 2025), we compared encoding models in the human scalp EEG dataset, time-locking features to either saccade or fixation, to assess how these predictors compare in their capacity to explain neural activity. First, we trained encoding models using only saccade-onset and only fixation-onset predictors to see which better predicts neural activity. Next, we trained another pair of models, this time comprising onset and amplitude locked to saccades or fixations, and we analyzed how much the predictions of neural activity improved with amplitude. Specifically, we measured how much the Pearson correlation between true and predicted responses increased when adding amplitude beyond the onset model, i.e., the change in correlation between the onset-only model and the onset+amplitude model. We then compared those increases between the saccade- and fixation-locked models. We then trained another pair of models comprising onset, amplitude, and novelty, locked to either saccades or fixations. As before, we measure how much predictions of neural activity improved with novelty by comparing the onset+amplitude model and the onset+amplitude+novelty model for both saccade- and fixation-locked events.

### Brain plots

Scalp EEG topoplots were constructed using the MNE topomap visualization and BioSemi64 montage (Gramfort, 2013). Contour lines are shown along interpolated constant values for each scalp map.

Human intracranial EEG brain plots were constructed using the electrode localization and plotting features in naplib-python (Mischler et al., 2023). Specifically, naplib can use Freesurfer-defined atlases to provide anatomical labels of electrodes based on FSAverage coordinates, as well as interpolate individual values onto the brain and summarize within atlas regions (Fischl, 2012). Here, we used the Desikan-Killiany atlas for displaying the percent electrodes predicted significantly and electrode latency, as this is a relatively coarse atlas showing broad effects (Desikan et al., 2006). The latency values for each region were obtained by taking the average latency of all electrodes labeled in that region. We also used the Glasser atlas for displaying the modulation of individual electrodes over the brain, as this is a more fine-grained atlas that allows the identification of more relevant subregions (Glasser et al., 2016). The modulation values for each region were obtained by first interpolating the modulation value of each electrode onto the neighboring area using inverse distance weighting (max. distance 8mm) and then taking the average over all points in each region.

## Supplementary Material

Supplement 1

## Significance statement

Primate vision relies on eye movements to sample the world, yet how the brain integrates high-level meaning across these "snapshots" remains a mystery. By combining deep-learning models with massive electrophysiological datasets from humans and macaques, we show that the brain responds to changes in semantic content between fixations. We identified a "semantic novelty" signal that modulates neural activity across the primate cortex, appearing in frontal areas before a saccade is completed. These findings suggest visual processing is not a series of independent glimpses, but a continuous process integrating foveal information at the semantic level. This work bridges biological vision and artificial intelligence, providing a new framework for understanding how the brain maintains a coherent understanding of a dynamic world.
