# Supplementary material for: During natural vision, semantic novelty modulates fixation-related processing in primate cortex
Source: bioRxiv. 2026 May 14:2026.03.18.712708. Originally published 2026 Mar 20. Preprint. [Version 2] doi: 10.64898/2026.03.18.712708 (PMC13015500; doi:10.64898/2026.03.18.712708)
Supplement: Supplement 1 [file media-1.pdf]

Supplemental Figures

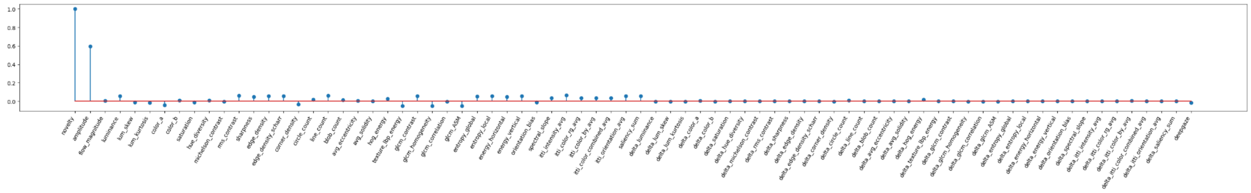

Figure S1. Correlation on novelty with other visual features. Novelty is only strongly correlated with saccade amplitude, but a small number of features related to luminance also show a small correlation.

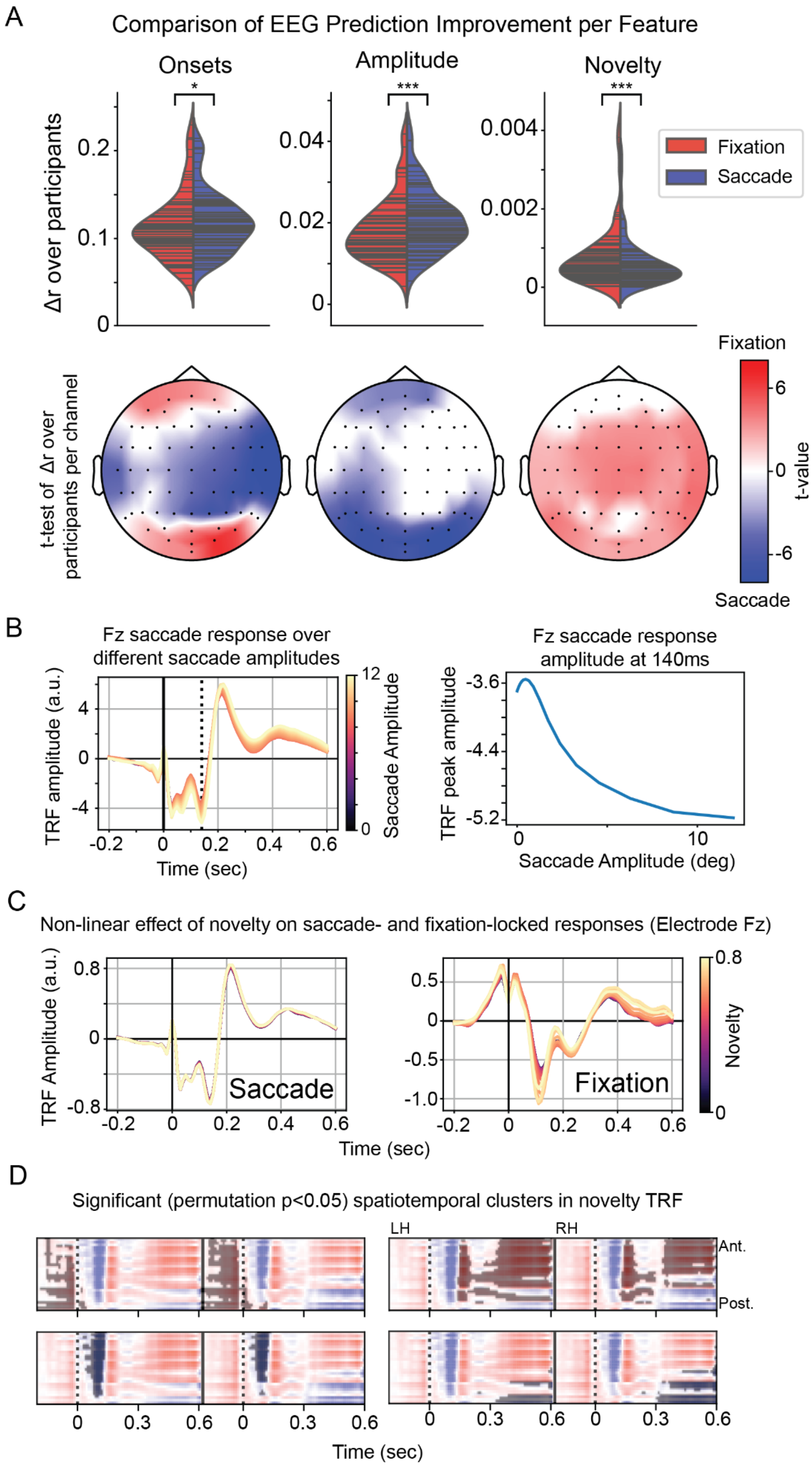

Figure S2. Modulation of scalp EEG potentials. A) Left: Saccade onsets capture slightly more variance than fixation onsets (*: $p<0.05$), and different electrodes are better explained by saccades vs fixations. Center: Amplitude locked to saccade onset captures more variance than

amplitude locked to fixation onset (***: $p<0.001$), and this effect is consistent across the scalp. Right: Novelty locked to fixation onset captures more variance than novelty locked to saccade onset, with amplitude regressed out (***: $p<0.001$), and this effect is consistent across the scalp. B) Saccade amplitude responses modeled with B-splines display a non-linear response with saccade amplitude.

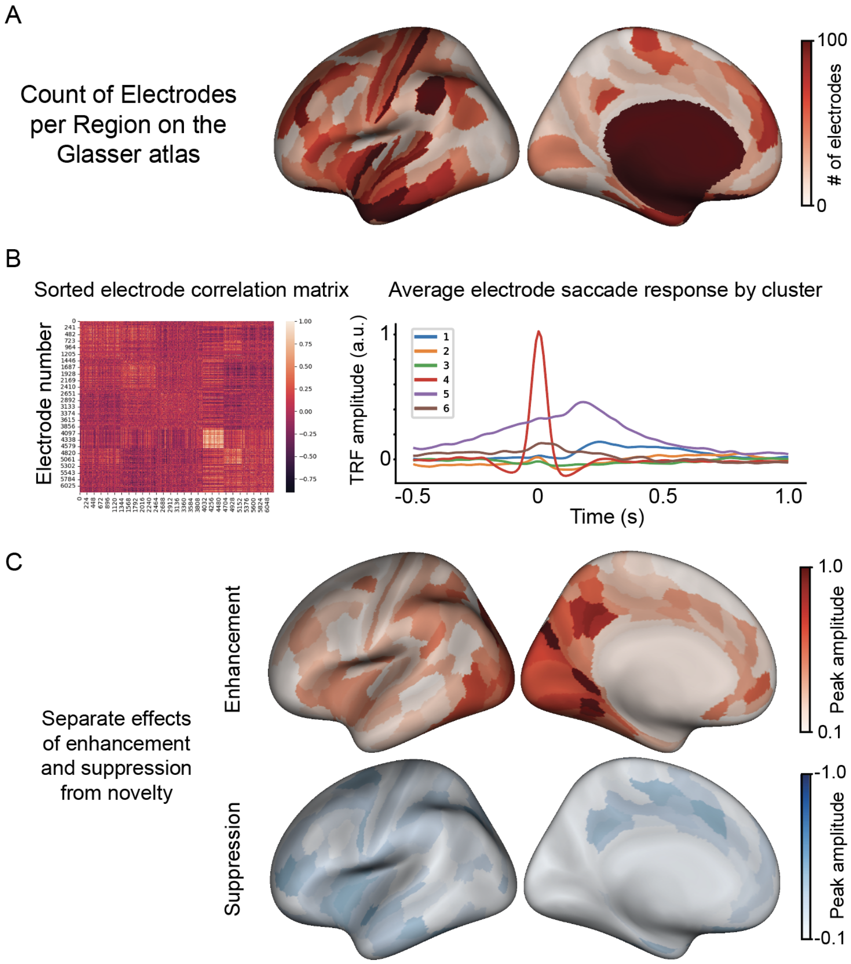

Figure S3. Human intracranial EEG coverage, electrode response clusters, and novelty enhancement vs suppression. A) Brain plot showing the total number of electrodes measured from each region of the Glasser atlas in the movie-watching dataset. B) Correlation matrix of electrode saccade response, sorted by clusters (left). Electrode saccade response, averaged within each cluster. Cluster 4 was identified as saccadic spike artifacts and removed from analysis. C) Distinct novelty modulations by enhancements and suppression over cortex. Responses are dominantly enhancing.

Energy	Color	Texture	Shape	Other
**Luminance**	Average a* color	Orientation bias	Hough line count	**Optical flow magnitude**
Luminance skew	Average b* color	**Spectrum slope**	Hough circle count	DeepGaze III saliency
Luminance kurtosis	Average saturation	Histogram of oriented gradients energy	Blob count	**Saccade amplitude**
Michelson contrast	Hue diversity	Local binary pattern energy	Average blob solidity	**Semantic novelty**
RMS contrast	RG color saliency	Sharr edge density	Average blob eccentricity	
Sharpness	BY color saliency	Sobel edge density		
Vertical energy	Total color saliency	GLCM contrast		
Horizontal energy		GLCM homogeneity		
Global entropy		GLCM correlation		
Local entropy		GLCM angular second moment		
Intensity-based saliency		Corner density		
Orientation-based saliency				
Total saliency				

Table S1. Visual features of fixation patches tested for correlation, with features modeled with EEG shown in bold.
