# Supplementary material for: During natural vision, semantic novelty modulates fixation-related processing in primate cortex
Source: bioRxiv. 2026 Mar 20:2026.03.18.712708. Preprint. [Version 1] doi: 10.64898/2026.03.18.712708 (PMC13015500; doi:10.64898/2026.03.18.712708)
Supplement: Supplementary file 1 [file media-1.pdf]

Supplemental Figures

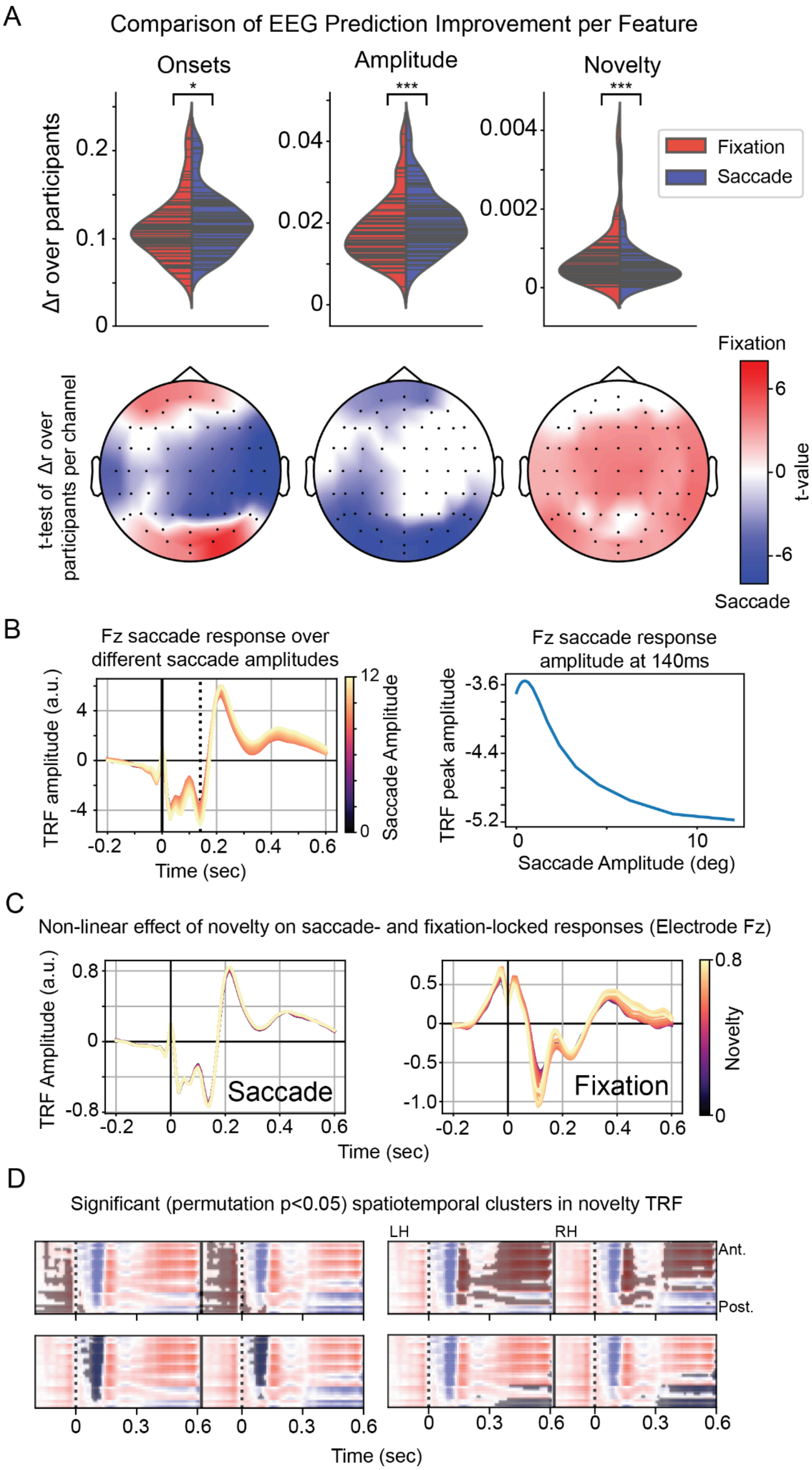

Supp. Fig. 1. Modulation of scalp EEG potentials. A) Left: Saccade onsets capture slightly more variance than fixation onsets (*: p<0.05), and different electrodes are better explained by saccades vs fixations. Center: Amplitude locked to saccade onset captures more variance than amplitude locked to fixation onset (***: p<0.001), and this effect is consistent across the scalp. Right: Novelty locked to fixation onset captures more variance than novelty locked to saccade onset, with amplitude regressed out (***: p<0.001), and this effect is consistent across the scalp. B) Saccade amplitude responses modeled with B-splines display a non-linear response with saccade amplitude.

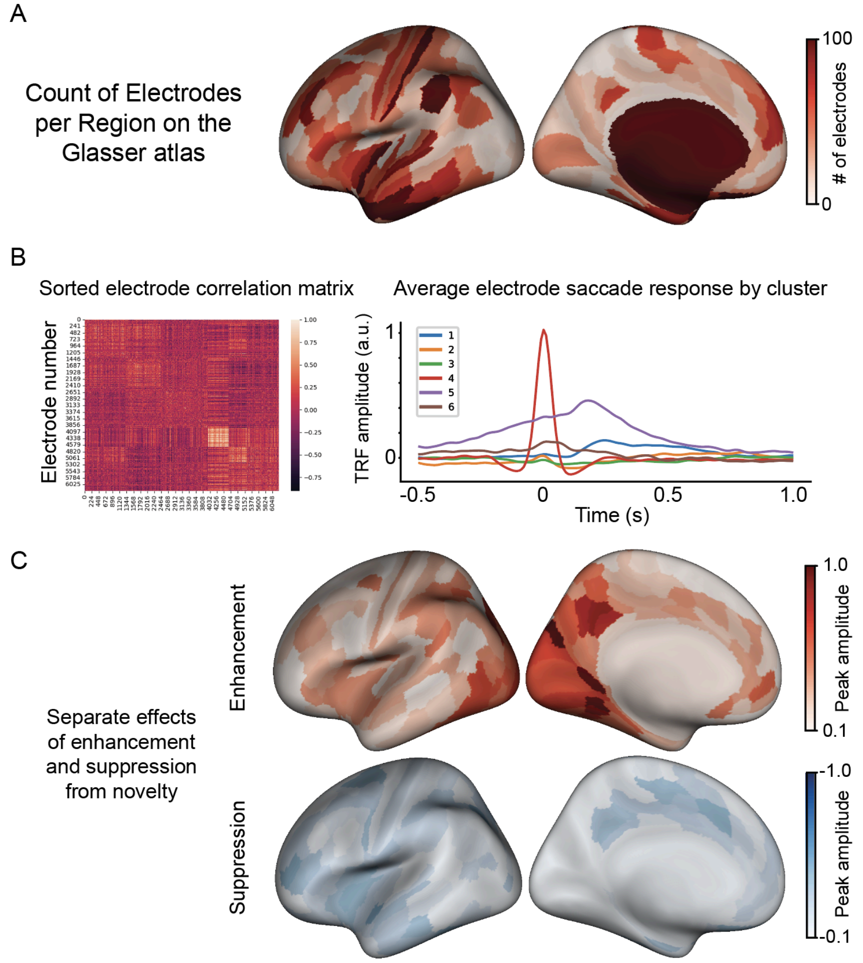

Figure S2. Human intracranial EEG coverage, electrode response clusters, and novelty enhancement vs suppression. A) Brain plot showing the total number of electrodes measured from each region of the Glasser atlas in the movie-watching dataset. B) Correlation matrix of electrode saccade response, sorted by clusters (left). Electrode saccade response, averaged within each cluster. Cluster 4 was identified as saccadic spike artifacts and removed from analysis. C) Distinct novelty modulations by enhancements and suppression over cortex. Responses are dominantly enhancing.
